# Supplementary figures and images for: Maximum trunk tip force assessment related to trunk position and prehensile ’fingers’ implication in African savannah elephants
Source: PLoS One. 2024 May 14;19(5):e0301529. doi: 10.1371/journal.pone.0301529 (PMC11093316; doi:10.1371/journal.pone.0301529)

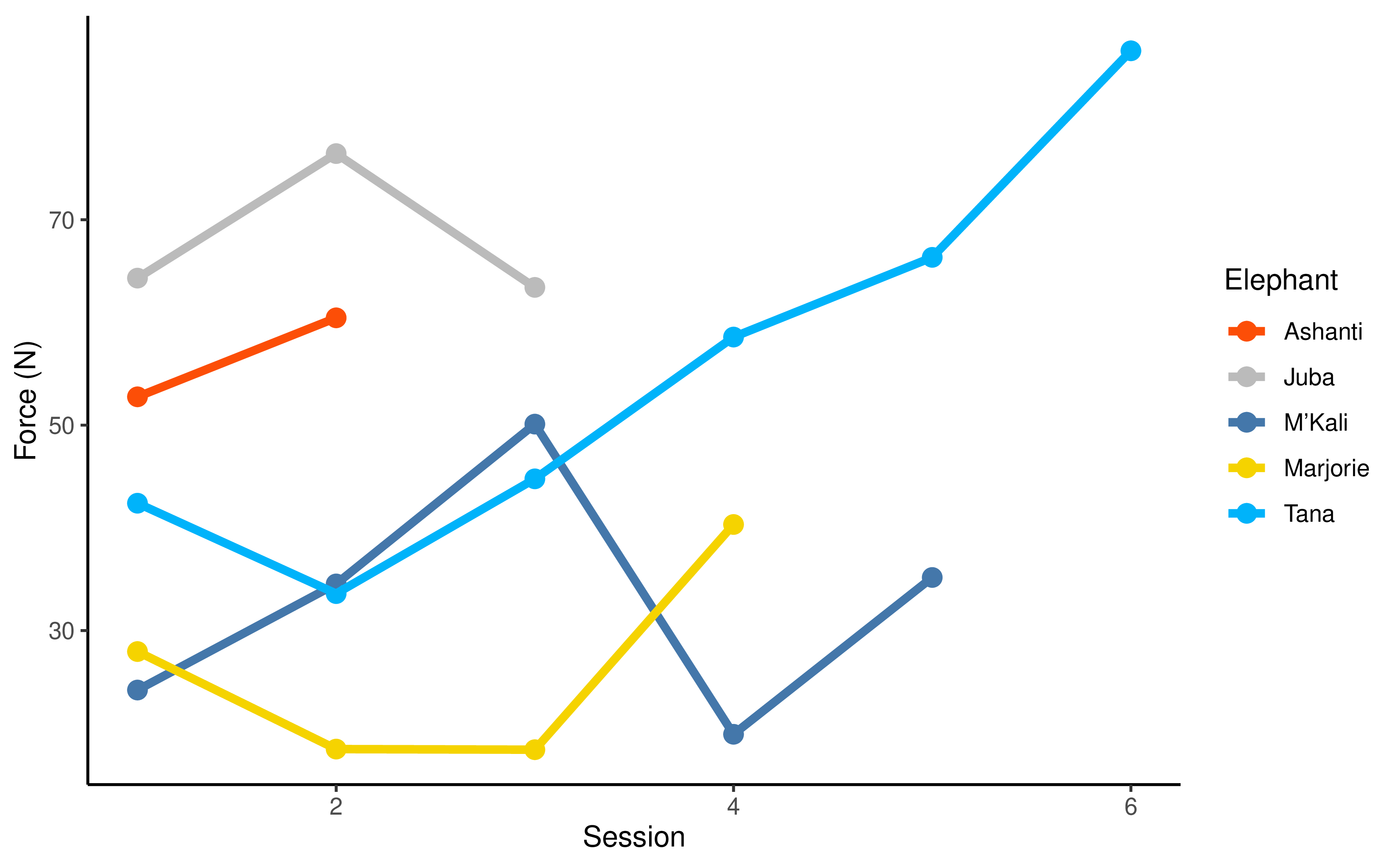

Supplement: S1 Fig — The force is expressed in Newton (N). Each colour represents an elephant: orange for Ashanti, grey for Juba, dark blue for M’Kali, yellow for Marjorie and light blue for Tana. (TIF) [file pone.0301529.s001.tif]

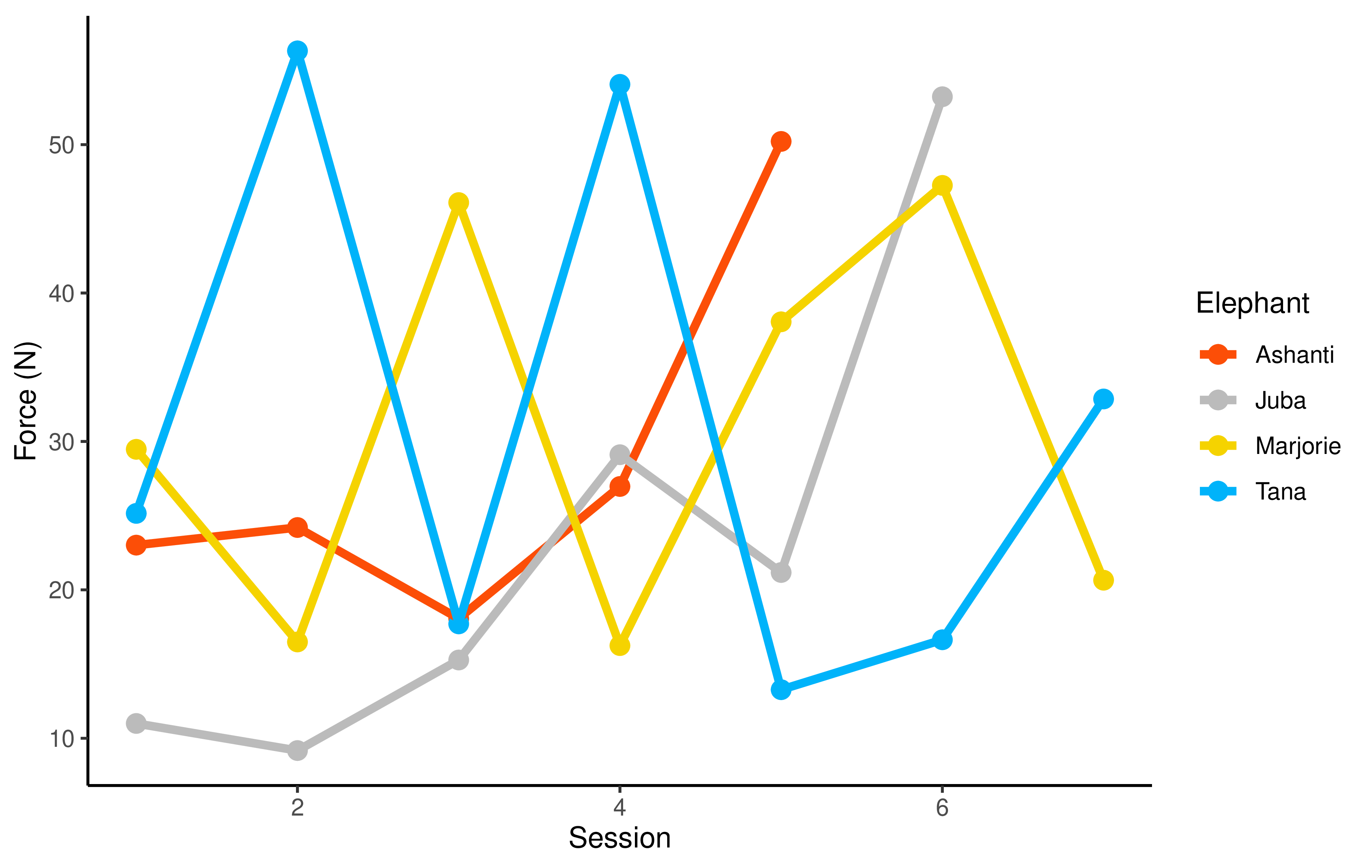

Supplement: S2 Fig — The force is expressed in Newton (N). Each colour represents an elephant: orange for Ashanti, grey for Juba, yellow for Marjorie and light blue for Tana. (TIF) [file pone.0301529.s002.tif]

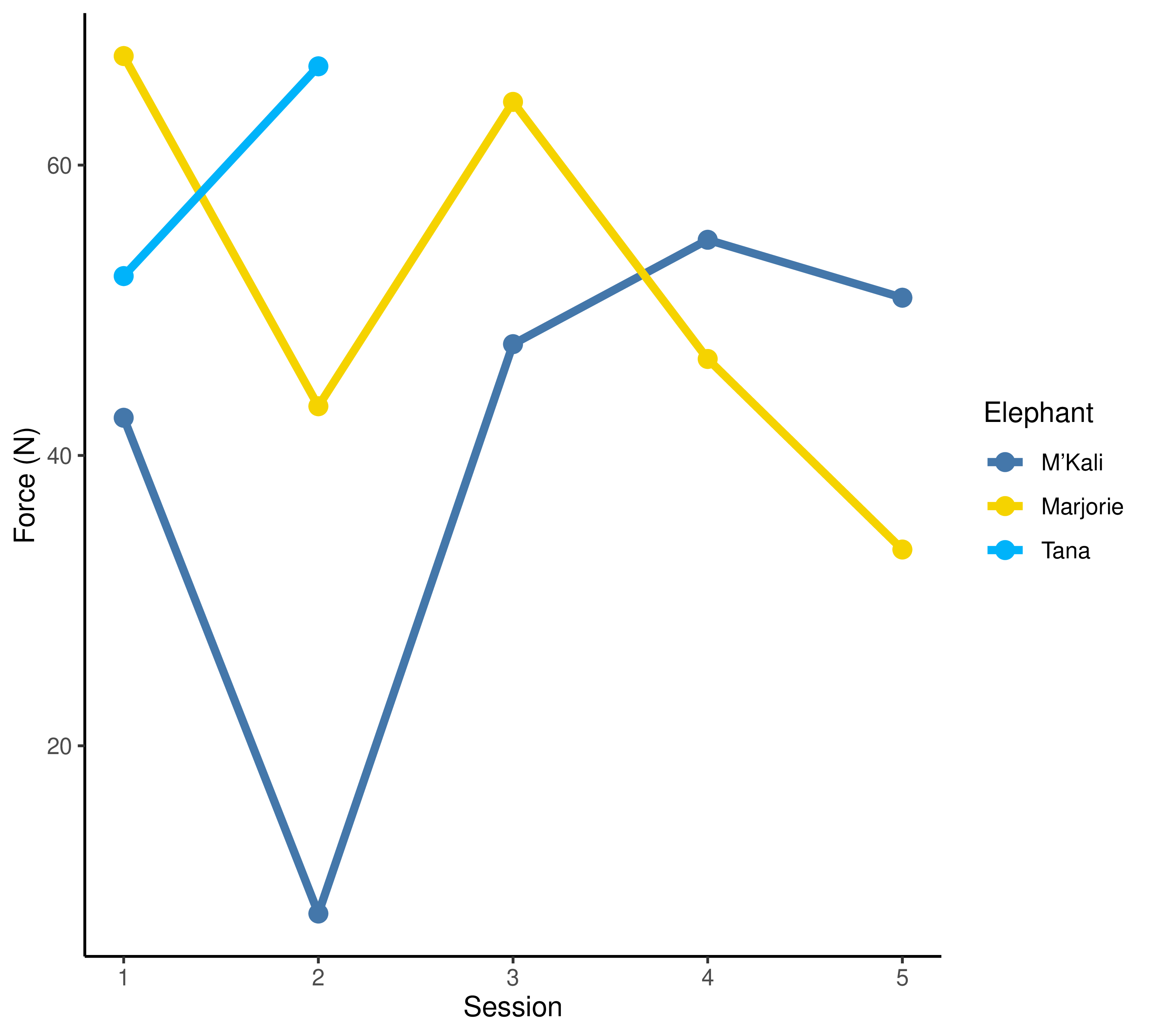

Supplement: S3 Fig — The force is expressed in Newton (N). Each colour represents an elephant: dark blue for M’Kali, yellow for Marjorie and light blue for Tana. (TIF) [file pone.0301529.s003.tif]

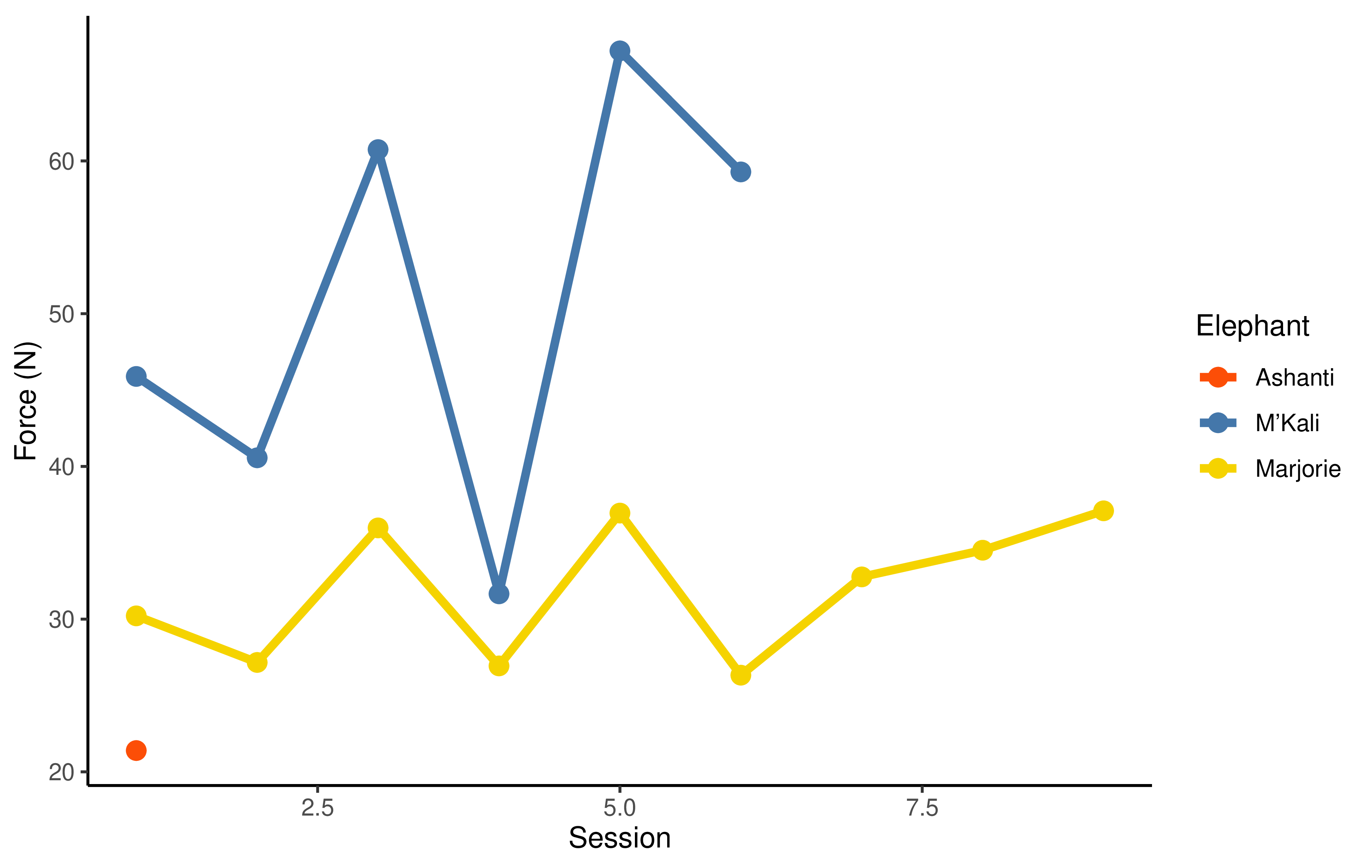

Supplement: S4 Fig — The force is expressed in Newton (N). Each colour represents an elephant: orange for Ashanti, dark blue for M’Kali and yellow for Marjorie. (TIF) [file pone.0301529.s004.tif]

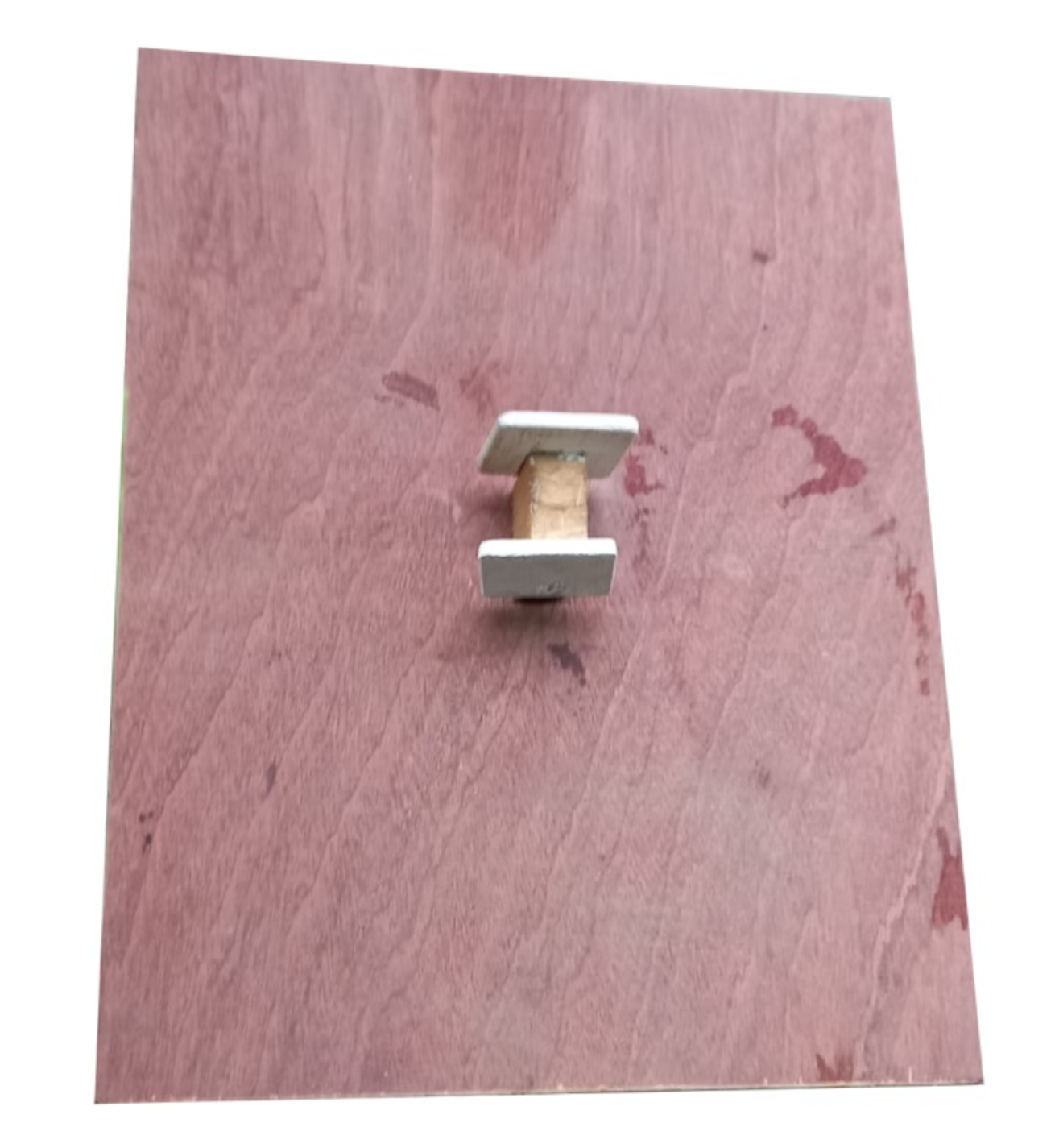

Supplement: S5 Fig — (TIF) [file pone.0301529.s005.tif]
