# Supplementary material for: Maximum trunk tip force assessment related to trunk position and prehensile ’fingers’ implication in African savannah elephants
Source: PLoS One. 2024 May 14;19(5):e0301529. doi: 10.1371/journal.pone.0301529 (PMC11093316; doi:10.1371/journal.pone.0301529)
Supplement: S1 Table — (DOCX) [file pone.0301529.s006.docx]

|  | **Unbent** | | **Bent** | |
| --- | --- | --- | --- | --- |
|  | **Vertical sensors** (N) | **Horizontal sensors** (N) | **Vertical sensors** (N) | **Horizontal sensors** (N) |
| **Tana** | **86.3** | **55.9** | **67.7** | / |
| **Juba** | 76.5 | 53 | / | / |
| **Ashanti** | 60.8 | 50 | / | 21.6 |
| **M’Kali** | 50 | / | 54.9 | **66.7** |
| **Marjorie** | 40.2 | 47.1 | 66.7 | 37.3 |
