# Supplementary material for: Maximum trunk tip force assessment related to trunk position and prehensile ’fingers’ implication in African savannah elephants
Source: PLoS One. 2024 May 14;19(5):e0301529. doi: 10.1371/journal.pone.0301529 (PMC11093316; doi:10.1371/journal.pone.0301529)
Supplement: S2 Table — (DOCX) [file pone.0301529.s007.docx]

|  | **Unbent** | | **Bent** | |
| --- | --- | --- | --- | --- |
|  | **Vertical sensors** (N) | **Horizontal sensors** (N) | **Vertical sensors** (N) | **Horizontal sensors** (N) |
| **Dorsal finger** | **60.2** | 34.4 | **58** | 37.6 |
| **Ventral finger** | 30.1 | **38.1** | 18.6 | **41.7** |
