# Supplementary material for: Maximum trunk tip force assessment related to trunk position and prehensile ’fingers’ implication in African savannah elephants
Source: PLoS One. 2024 May 14;19(5):e0301529. doi: 10.1371/journal.pone.0301529 (PMC11093316; doi:10.1371/journal.pone.0301529)
Supplement: S3 Table — (DOCX) [file pone.0301529.s008.docx]

|  | Vertical sensors | | | Horizontal sensors | | | **All sensors’ orientations** | |
| --- | --- | --- | --- | --- | --- | --- | --- | --- |
|  | Pinch grasps | | Total grasp | Pinch grasps | | Total grasps | **Total pinches** | **Total grasps** |
|  | Straight trunk | Bent trunk |  | Twisted trunk | Twisted and bent trunk |  |  |  |
| Tana | 66 | 4 | 80 | 16 | 0 | 140 | **86** | **220** |
| M’Kali | 37 | 38 | 80 | 0 | 37 | 197 | **112** | **277** |
| Ashanti | 7 | 0 | 54 | 17 | 2 | 94 | **26** | **148** |
| Juba | 43 | 0 | 43 | 17 | 0 | 90 | **60** | **133** |
| Marjorie | 9 | 31 | 65 | 25 | 29 | 169 | **94** | **234** |
| Total | 162 | 73 | 322 | 75 | 68 | 690 | **378** | **1012** |
