# Supplementary material for: Maximum trunk tip force assessment related to trunk position and prehensile ’fingers’ implication in African savannah elephants
Source: PLoS One. 2024 May 14;19(5):e0301529. doi: 10.1371/journal.pone.0301529 (PMC11093316; doi:10.1371/journal.pone.0301529)
Supplement: Attachment — Submitted filename: Rebuttal_letter.docx [file pone.0301529.s009.docx]

*January 22nd, 2023*

*Dear Academic Editor of PLOS ONE,*

*We would like to thank the reviewers for their time and precious comments on our manuscript. We have edited the paper in the light of their remarks.*

*We hope that this corrected manuscript is now suitable for publication.*

*On behalf of all authors.*

*Pauline Costes*

*PhD Student at the Muséum National d'Histoire Naturelle*

**Academic Editor**

Dear Dr. Costes,

Thank you for submitting your manuscript to PLOS ONE. After careful consideration, we feel that it has merit but does not fully meet PLOS ONE’s publication criteria as it currently stands. Therefore, we invite you to submit a revised version of the manuscript that addresses the points raised during the review process.

Please submit your revised manuscript by Jan 19 2024 11:59PM. If you will need more time than this to complete your revisions, please reply to this message or contact the journal office at plosone@plos.org. When you're ready to submit your revision, log on to <https://www.editorialmanager.com/pone/> and select the 'Submissions Needing Revision' folder to locate your manuscript file.

Please include the following items when submitting your revised manuscript:

- A rebuttal letter that responds to each point raised by the academic editor and reviewer(s). You should upload this letter as a separate file labeled 'Response to Reviewers'.
- A marked-up copy of your manuscript that highlights changes made to the original version. You should upload this as a separate file labeled 'Revised Manuscript with Track Changes'.
- An unmarked version of your revised paper without tracked changes. You should upload this as a separate file labeled 'Manuscript'.

If you would like to make changes to your financial disclosure, please include your updated statement in your cover letter. Guidelines for resubmitting your figure files are available below the reviewer comments at the end of this letter.

If applicable, we recommend that you deposit your laboratory protocols in [protocols.io](http://protocols.io) to enhance the reproducibility of your results. Protocols.io assigns your protocol its own identifier (DOI) so that it can be cited independently in the future. For instructions see: <https://journals.plos.org/plosone/s/submission-guidelines#loc-laboratory-protocols>. Additionally, PLOS ONE offers an option for publishing peer-reviewed Lab Protocol articles, which describe protocols hosted on protocols.io. Read more information on sharing protocols at https://plos.org/protocols?utm_medium=editorial-email&utm_source=authorletters&utm_campaign=protocols.

We look forward to receiving your revised manuscript.

Kind regards,

Monika Błaszczyszyn
Academic Editor
PLOS ONE

*→ Thank you very much for this feedback on our paper. We hope this corrected version is now suitable for publication in PLOS ONE.*

**Journal requirements:**

When submitting your revision, we need you to address these additional requirements.

1. Please ensure that your manuscript meets PLOS ONE's style requirements, including those for file naming. The PLOS ONE style templates can be found at

<https://journals.plos.org/plosone/s/file?id=wjVg/PLOSOne_formatting_sample_main_body.pdf> and

<https://journals.plos.org/plosone/s/file?id=ba62/PLOSOne_formatting_sample_title_authors_affiliations.pdf>

*→ Thank you for this reminder, our manuscript meets PLOS ONE's style requirements.*

2. Note from Emily Chenette, Editor in Chief of PLOS ONE, and Iain Hrynaszkiewicz, Director of Open Research Solutions at PLOS: Did you know that depositing data in a repository is associated with up to a 25% citation advantage (<https://doi.org/10.1371/journal.pone.0230416>)? If you’ve not already done so, consider depositing your raw data in a repository to ensure your work is read, appreciated and cited by the largest possible audience. You’ll also earn an Accessible Data icon on your published paper if you deposit your data in any participating repository (<https://plos.org/open-science/open-data/#accessible-data>).

*→ We have deposited* *the raw data in the repository Open Science Framework. They are now freely available by following this link:* [*https://osf.io/85kj3/?view_only=d245827940504ad7b6f0461de2b1301b*](https://osf.io/85kj3/?view_only=d245827940504ad7b6f0461de2b1301b)

*We have also added the reference in the appropriate section. Costes, P., Delapré, A., Houssin, C., Mulot, B., Pouydebat, E., & Raphaël, C. (2023, December 7). Elephant pinching forces. Retrieved from osf.io/85kj3*

3. We note that the grant information you provided in the ‘Funding Information’ and ‘Financial Disclosure’ sections do not match.

When you resubmit, please ensure that you provide the correct grant numbers for the awards you received for your study in the ‘Funding Information’ section.

*→ Thank you for pointing out this error. We have updated our declaration for the* *'Financial Disclosure' section in the cover letter and the 'Funding Information' section in the submission form.*

*We would like to make the following changes to our financial statement in the 'Financial Disclosure' section: « This work was supported by grants IBEES 20SB403U7209, Association Sorbonne Université (EP) and CNRS 80 PRIME, Centre National de la Recherche Scientifique (EP), which we thank. The funders had no role in study design, data collection and analysis, decision to publish, or preparation of the manuscript. »*

4. We note that Figure 1 in your submission contain copyrighted images. All PLOS content is published under the Creative Commons Attribution License (CC BY 4.0), which means that the manuscript, images, and Supporting Information files will be freely available online, and any third party is permitted to access, download, copy, distribute, and use these materials in any way, even commercially, with proper attribution. For more information, see our copyright guidelines: [http://journals.plos.org/plosone/s/licenses-and-copyright](http://journals.plos.org/plosone/s/licenses-and-copyright).

We require you to either (1) present written permission from the copyright holder to publish these figures specifically under the CC BY 4.0 license, or (2) remove the figures from your submission:

a. You may seek permission from the original copyright holder of Figure 1 to publish the content specifically under the CC BY 4.0 license.

We recommend that you contact the original copyright holder with the Content Permission Form (<http://journals.plos.org/plosone/s/file?id=7c09/content-permission-form.pdf>) and the following text:

“I request permission for the open-access journal PLOS ONE to publish XXX under the Creative Commons Attribution License (CCAL) CC BY 4.0 (<http://creativecommons.org/licenses/by/4.0/>). Please be aware that this license allows unrestricted use and distribution, even commercially, by third parties. Please reply and provide explicit written permission to publish XXX under a CC BY license and complete the attached form.”

Please upload the completed Content Permission Form or other proof of granted permissions as an "Other" file with your submission.

In the figure caption of the copyrighted figure, please include the following text: “Reprinted from [ref] under a CC BY license, with permission from [name of publisher], original copyright [original copyright year].”

b. If you are unable to obtain permission from the original copyright holder to publish these figures under the CC BY 4.0 license or if the copyright holder’s requirements are incompatible with the CC BY 4.0 license, please either i) remove the figure or ii) supply a replacement figure that complies with the CC BY 4.0 license. Please check copyright information on all replacement figures and update the figure caption with source information. If applicable, please specify in the figure caption text when a figure is similar but not identical to the original image and is therefore for illustrative purposes only.

please specify in the figure caption text when a figure is similar but not identical to the original image and is therefore for illustrative purposes only.

*→ We realise that we have completed the form incorrectly during submission. Our Figure 1 has been created from scratch by the authors and the represented device is not the same. Only the design is similar (but not identical) to the original image. However, if authorisation is still required, we can make the request.* ***The apples in this figure were drawn by the authors.***

5. We notice that your supplementary figures are included in the manuscript file. Please remove them and upload them with the file type 'Supporting Information'. Please ensure that each Supporting Information file has a legend listed in the manuscript after the references list.

*→ Thank you for this clarification. We have removed the supplementary figures in the manuscript file and uploaded them with the file type 'Supporting Information'.*

**Reviewer #1**: Strong aspects:

The authors measure the force of the fingers of the trunk of several elephants and find out that one finger has stronger force than the other.

Weak aspects:

Although the findings are new the possible implications or applications of this particularity of the elephant trunk are not mentioned. I suggest to improve of the paper by giving possible reasons why the dorsal finger is more powerful. Also, a comparison with the human hand would be interesting.

*→ Thank you for your comments. As you advised, we discussed the implications of the difference in force between the two trunk fingers and added a comparison with the human hand which allows us to discuss another explanation for the difference in force between the two fingers,* *in addition to the morphological and anatomical differences already mentioned.*

*The following paragraph has been added to the discussion in the "Force distribution between the fingers" section (lines 342): “**In comparison, in humans, when the thumb and index finger pinch, the forces are not always equal. Depending on the surface, the thumb may be stronger than the index finger (thumb: 7.48±0.13 N vs index: 6.11±0.20 N), or both fingers may be equally strong (thumb: 6.32±0.14 N vs index: 6.08±0.14 N) (Dollahon et al, 2022). In addition, the directions of force vectors were dependent on the magnitude of the pinch force applied, especially at force levels below 2 N. The two force vectors were never observed to be perfectly opposed, whereby not intrinsically satisfying static equilibrium (Li et al., 2013). The same phenomenon could occur between the two fingers of the elephant's trunk, explaining the greater force of the dorsal finger compared to the ventral one.”*

*Dollahon, Devon, Seokchang Ryu, et Hangue Park. « Pinching Force Changes by Modulating the Interaction Gain Over the Fingertip ». IEEE Access 10 (2022): 9744‑49.* [*https://doi.org/10.1109/ACCESS.2022.3143837*](https://doi.org/10.1109/ACCESS.2022.3143837)*.*

*Li, Ke, Raviraj Nataraj, Tamara L. Marquardt, et Zong-Ming Li. « Directional Coordination of Thumb and Finger Forces during Precision Pinch ». PLOS ONE 8, n^o^ 11 (2013): e79400.* [*https://doi.org/10.1371/journal.pone.0079400*](https://doi.org/10.1371/journal.pone.0079400)*.*

Comments

The latin name for this specie of elephant is given twice: at the beginning of the paper and around line 200.

*→ Thank you for your comment, the second mention of "Loxodonta africana" on line 102 has been deleted.*

**Reviewer #2**: This manuscript measures the distal gripping force of female African savanna elephants through a cleverly designed experiment on captive individuals. The study is able to display interesting results related to maximum pinch force, as well as differences between the two fingers of the trunk. I enjoyed reading the manuscript but I do think that it requires more polishing before acceptance. Please see my comments below. I also suggest that the manuscript is proof read for the English, as some sections were either a little difficult to make sense of, or words such as "Indeed" are over-used. I hope that my comments will be helpful in improving your manuscript. Well done.

Please be careful with your changing of tenses within sections. I have pointed it out a couple times in the Methods but I see it happening in the Results as well.

*→ Thanks for the positive feedback, we've improved the English of this manuscript, notably by using the word "Indeed" less and by harmonizing the tenses used.*

Introduction

Page 3, line 48: Scientific name for Asian elephants can be mentioned earlier in the Intro when you first mention them.

*→ The mention of "Elephas maximus" on line 48 has been moved to line 40.*

Methods

Page 5, line 101: Change to "and then in April 2023."

*→ The sentence has been modified, thank you.*

Page 5, line 102: no need to re-mention the scientific name here.

*→ Thank you for your comment, the second mention of "Loxodonta africana" on line 102 has been deleted.*

Table 1: Rather add a column heading for birth year and a column heading for birth location. Then the Origin column can be divided into these 2 columns to make for easier reading.

*→ The "Origin" column has been split in two to create two separate columns for "Birth location" and "Birth year". The table is now easier to read.*

Page 5, line 106: Rather say "In this study, we observed elephants in an indoor setting."

*→ The sentence has been modified, thank you.*

Page 5, lines 106-108: Please rephrase the English of the second sentence of this paragraph.

*→ The sentence “Every morning, one to three elephants isolated were groomed and/or trained to facilitate any future medical interventions in a pen dedicated to daily care and medical training.” was replaced by the following: "* *Every morning,* *time is devoted to medical training: one to three elephants are isolated in an enclosure dedicated to daily care and grooming, to facilitate any future medical intervention."*

Page 6, lines 118-127: Stick to the same tense for the paragraph. Here it changes from present to past tense. The same goes for the "The Course of the Experiment" paragraph.

*→ All the verbs in the paragraphs "Functioning of the device" and "The Course of the experiment" are now in the past tense.*

Page 6, line 122: Change to "pinch hard enough to pass..."

*→ The sentence has been modified, thank you.*

Page 8, lines 180-183: Please combine these sentences with a comma.

*→ The sentences have been combined to become:* *“For example, if two elephants A and B pinched the sensors 6 and 10 times respectively, the comparison will be made with the 6 force measurements of elephant A and the 6 highest force measurements of elephant B.”*

Page 9, line 192: Rather say "as previously mentioned."

*→ The sentence has been modified, thank you.*

Table 2: I think that Table 2 can be made more clear to the reader. Look at splitting the 3rd and 4th columns into subheadings where the value is placed below. The current state makes it difficult to understand and appreciate the variation of the recorded values.

*→ Table 2 has been clarified and we hope it will be easier to read.*

Results

Page 10, line 204: Rather say "summarised" instead of "summed up"

*→ The sentence has been modified, thank you.*

Page 11, lines 216-218: Even though the p values are shown in the graph, please provide the full statistics here and the average force values.

Page 11, lines 228-229: Give the average values if you can here.

*→ Thank you for pointing out these omissions, we have provided the full statistics for each test and all the mean values mentioned in the results section.*

Page 11, line 230: What do you mean more important? Do you mean greater?

*→* *Thank you for pointing out that imprecision, we did mean greater. The sentence has been modified.*

Page 12, lines 241-244: Here in brackets you can actually put what the maximum bent and unbent values were. E.g. - (??N bent vs ??N unbent).

*→ The maximum force values have been added to the sentence. The new sentence is as follows: “However, the results also showed that the maximum pinch force on vertical sensors was higher when the trunk was unbent* *(67.7 N bent vs 86.3 N unbent), while the maximum pinch force on horizontal sensors was higher when the trunk was bent (**66.7 N bent vs 55.9 N unbent; S1 Table).”*

Figure 6 - Instead of placing the full p-values for all of your figures, rather use the *, **, *** code system representing p<0.05; 0.01; 0.001. It will help neaten the figures.

*→ We have used the *, **, *** code system for Figures 4, 5 and 6. We have also modified the legends.*

Page 13, lines 272-273: Please correct the English of this sentence.

*→ The sentence has been corrected* *and simplified. The new sentence is as follows: “The dorsal finger always pressed harder on the vertical sensor than the ventral finger during pinches (on average, dorsal: 28.74 N vs ventral: 6.67 N; Fig 6).*

Page 14, line 285: Again, rather rephrase the word "important" to something of increased or decreased

*→ Thank you for pointing out that imprecision, we replaced “was more important” with “increased” in this sentence.*

Discussion

Page 14, line 294: The lower force recorded by your study? Please be specific.

*→ Yes, we did mean the lower force recorded by our study. The sentence has been completed, thank you.*

Page 14, line 299: Remove "we know" - the audience may not know.

*→ We removed “we know” which was not appropriate.*

Page 14, lines 304-305: Remove "On one hand" and then instead of saying "Our hypothesis was wrong", rather add "disproving our hypothesis" to the end of the first sentence.

*→ The sentence has been modified, thank you.*

Page 15, line 313: Remove "On the other hand"

*→ The sentence has been modified, thank you.*

Page 15, line 319: The word "Indeed" is used a lot in the Discussion. Try substitute it for other words of the same meaning.

*→ We thank you for your comment and have taken it into account.*

Page 15, line 327: Remove "All pinches combined"

*→ The sentence has been modified, thank you.*

Page 15, line 328: Change is to was

*→ The sentence has been modified, thank you.*

Page 16, lines 339-355: Please try and rewrite this paragraph in a more concise manner. It feels very long-winded and I am sure could be reduced into a more concise description.

*→* *The paragraph has been rewriting:* *"However, the force distribution between the fingers was related to the trunk position. On one hand, torsion reduced the difference in force between the fingers and increased the number of pinches in which the ventral finger was the strongest. Indeed, the torsion reduced the dorsal finger's maximum force and increased the ventral finger's maximum force. On the other hand, bending reduced the maximum force of the dorsal and ventral fingers and the difference in force between the fingers was similar on average between the unbent and bent trunk with the vertical sensors. With the horizontal sensors, the bending, combined with torsion, increased the difference in force between the fingers and the number of pinches in which the ventral finger was the strongest. The bending increased the maximum force of the dorsal and ventral fingers but not in the same proportions. The muscles used to bend the trunk and to pinch are the same, i.e. the longitudinal and radial groups. The oblique muscles are used to twist the trunk and are also present in the ventral finger, more aligned longitudinally than in the rest of the trunk. It is possible that the contraction of these muscles, which twist the trunk, indirectly increases the force of the ventral finger.”*

I noticed that you spoke about robotics extensively in the Introduction, which lead me to believe that the Discussion would loop back to this, however, robotics is never mentioned in the Discussion. I suggest either cutting down its portion in the Intro drastically, or show here in the Discussion how what you have found relates back to robotics.

*→* *Robotics seems to us to be an interesting aspect of this work and has not been sufficiently addressed in the discussion. We have therefore added the following paragraph at the end of the discussion: “Desirable properties of soft robot actuators include infinite degree of freedom, large deformation, high compliance and relatively large output force (**for a review on soft robotic grippers see Shintake et al., 2018*)*. Currently, the reliability of gripping small objects is often not high due to the small contact area (Liu et al., 2021). The reliability of pinch gripping has been improved by creating a two-finger soft-robotic gripper with modules of different heights (Liu et al., 2021). However, their gripper and all other existing two-finger soft-robotic grippers have a symmetrical constitution with fingers of the same shape and deploying the same force (example: Liu et al., 2018; Liu et al., 2021; Teeple et al., 2020).* *There are already asymmetrical robots mimicking the trunk body, similar to the non-homogeneity of the elephant trunk during stretching, which is not due to muscle but to asymmetries in the properties of the skin (Sanchez et al., 2023; Schulz et al., 2022, 2023). This study also highlights the benefits of asymmetries in terms of shape and force between the two fingers, which could lead to more versatile robotic grippers that are effective in grasping without having to deploy greater force."*

*Liu, Chih-Hsing, Ta-Lun Chen, Chen-Hua Chiu, Mao-Cheng Hsu, Yang Chen, Tzu-Yang Pai, Wei-Geng Peng, et Yen-Pin Chiang. « Optimal Design of a Soft Robotic Gripper for Grasping Unknown Objects ». Soft Robotics 5, no 4 (28 août 2018): 452 65.* [*https://doi.org/10.1089/soro.2017.0121*](https://doi.org/10.1089/soro.2017.0121)*.*

*Liu, Shoufeng, Fujun Wang, Zhu Liu, Wei Zhang, Yanling Tian, et Dawei Zhang. « A Two-Finger Soft-Robotic Gripper With Enveloping and Pinching Grasping Modes ». IEEE/ASME Transactions on Mechatronics 26, n^o^ 1 (février 2021): 146‑55.* [*https://doi.org/10.1109/TMECH.2020.3005782*](https://doi.org/10.1109/TMECH.2020.3005782)*.*

Sanchez, Vanessa, Kausalya Mahadevan, Gabrielle Ohlson, Moritz A. Graule, Michelle C. Yuen, Clark B. Teeple, James C. Weaver, James McCann, Katia Bertoldi, et Robert J. Wood. « 3D Knitting for Pneumatic Soft Robotics ». *Advanced Functional Materials* 33, n^o^ 26 (2023): 2212541. <https://doi.org/10.1002/adfm.202212541>.

Schulz, Andrew K., Madeline Boyle, Colin Boyle, Sophia Sordilla, Catalina Rincon, Scott Hooper, Catie Aubuchon, Joy S. Reidenberg, Claire Higgins, et David L. Hu. « Skin wrinkles and folds enable asymmetric stretch in the elephant trunk ». *Proceedings of the National Academy of Sciences* 119, n^o^ 31 (2 août 2022): e2122563119. <https://doi.org/10.1073/pnas.2122563119>.

Schulz, Andrew K., Nikole Schneider, Margaret Zhang, et Krishma Singal. « A Year at the Forefront of Hydrostat Motion ». *Biology Open* 12, n^o^ 8 (15 août 2023): bio059834. <https://doi.org/10.1242/bio.059834>.

Shintake, Jun, Vito Cacucciolo, Dario Floreano, et Herbert Shea. « Soft Robotic Grippers ». *Advanced Materials* 30, n^o^ 29 (2018): 1707035. <https://doi.org/10.1002/adma.201707035>.

Teeple, Clark B, Theodore N Koutros, Moritz A Graule, et Robert J. Wood. « Multi-Segment Soft Robotic Fingers Enable Robust Precision Grasping ». The International Journal of Robotics Research 39, no 14 (1 décembre 2020): 1647 67. <https://doi.org/10.1177/0278364920910465>.
